# Increase in Resistance to Extended-Spectrum Cephalosporins in *Salmonella* Isolated from Retail Chicken Products in Japan

**DOI:** 10.1371/journal.pone.0116927

**Published:** 2015-02-02

**Authors:** Tamie Noda, Koichi Murakami, Yoshiki Etoh, Fuyuki Okamoto, Jun Yatsuyanagi, Nobuyuki Sera, Munenori Furuta, Daisuke Onozuka, Takahiro Oda, Tetsuo Asai, Shuji Fujimoto

**Affiliations:** 1 Department of Health Sciences, Faculty of Medical Sciences, Kyushu University, 3-1-1 Maidashi, Higashi-ku, Fukuoka 812-8582, Japan; 2 Division of Pathology and Bacteriology, Department of Health Science, Fukuoka Institute of Health and Environmental Sciences, 39 Mukaizano, Dazaifu, Fukuoka 818-0135, Japan; 3 Akita Prefectural Institute of Public Health, 6-6 Sensyukubota, Akita 010-0874, Japan; 4 Nakamura Gakuen University Junior College, 5-7-1 Befu, Jonan-ku, Fukuoka 814-0198, Japan; 5 Department of Health Care Administration and Management, Kyushu University Graduate School of Medical Sciences, 3-1-1 Maidashi, Higashi-ku, Fukuoka 812-8582, Japan; 6 Animal Infectious Disease Control, United Graduate School of Veterinary Sciences, Gifu University, 1-1 Yanagido, Gifu 501-1193, Japan; Beijing Institute of Microbiology and Epidemiology, CHINA

## Abstract

Extended-spectrum β-lactamase (ESBL)-producing *Salmonella* are one of the most important public health problems in developed countries. ESBL-producing *Salmonella* strains have been isolated from humans in Asian countries neighboring Japan, along with strains harboring the plasmid-mediated extended-spectrum cephalosporin (ESC)-resistance gene, *ampC* (pAmpC). However, only a few studies have investigated the prevalence of ESC-resistant *Salmonella* in chicken products in Japan, which are the main vehicle of *Salmonella* transmission. The aim of this study was to investigate the prevalence of ESBL-producing, pAmpC-harboring, or carbapenem-resistant *Salmonella* in chicken products in Japan. In total, 355 out of 779 (45.6%) chicken product samples collected from 1996–2010 contained *Salmonella*, resulting in 378 distinct isolates. Of these isolates, 373 were tested for resistance to ESCs, cephamycins, or carbapenems. Isolates that showed resistance to one or more of these antimicrobials were then examined by PCR and DNA sequence analysis for the presence of the *bla*_CMY_, *bla*_CTX-M_, *bla*_TEM_, and *bla*_SHV_ resistance genes. Thirty-five resistant isolates were detected, including 26 isolates that contained pAmpC (*bla*_CMY-2_), and nine ESBL-producing isolates harboring *bla*_CTX-M_ (n = 4, consisting of two *bla*_CTX-M-2_ and two *bla*_CTX-M-15_ genes), *bla*_TEM_ (n = 4, consisting of one *bla*_TEM-20_ and three *bla*_TEM-52_ genes), and *bla*_SHV_ (n = 1, *bla*_SHV-12_). All pAmpC-harboring and ESBL-producing *Salmonella* isolates were obtained from samples collected after 2005, and the percentage of resistant isolates increased significantly from 0% in 2004 to 27.9% in 2010 (*P* for trend = 0.006). This increase was caused in part by an increase in the number of *Salmonella enterica* subsp. *enterica* serovar Infantis strains harboring an approximately 280-kb plasmid containing *bla*_CMY-2_ in proximity to IS*Ecp1*. The dissemination of ESC-resistant *Salmonella* containing plasmid-mediated *bla*_CMY-2_ in chicken products indicates the need for the development of continuous monitoring strategies in the interests of public health.

## Introduction

*Salmonella* infection remains a significant public health concern worldwide. In many countries, the incidence of human infections caused by extended-spectrum cephalosporin (ESC)-resistant *Salmonella* has increased dramatically [1,2]. ESCs are administrated to treat salmonellosis caused by fluoroquinolone-resistant *Salmonella*, or in children and infants [3,4]. However, ESC-resistance genes such as *bla*_CTX-M_, *bla*_TEM_, and *bla*_SHV_, which contribute to extended-spectrum β-lactamase (ESBL) production, have been detected in *Salmonella* isolated from humans in Asian countries neighboring Japan [5,6]. Some of these isolates also contain plasmid-mediated *ampC* (pAmpC), another ESC-resistance gene [7]. In Japan, ESC-resistant *Salmonella* containing pAmpC or ESBL genes, such as *bla*_CTX-M-14_ and *bla*_CTX-M-15_, have been isolated from humans [8,9].

ESC-resistant *Salmonella* have also been isolated from food-producing animals and their products in many countries [10,11]. Reports from Japan over the last decade show that these isolates are especially prevalent in chickens [12]. Moreover, ESBL genes such as *bla*_CMY_, *bla*_CTX-M_, *bla*_TEM_, and *bla*_SHV_ have been detected in *Salmonella* from food-producing-animal products in other Asian countries, and throughout the rest of the world [11,13]. In Japan, however, only a few studies have investigated the prevalence of ESC-resistant *Salmonella* in chicken products [14,15].

Because of the public health importance of these resistant salmonellae, it is imperative to determine their prevalence in chicken products in Japan. Therefore, the aim of this study was to investigate the prevalence of β-lactam antibiotic (cephalosporins or carbapenems) resistance in *Salmonella* from chicken products in Japan using molecular detection of ESBL genes and pAmpC.

## Materials and Methods

### Isolates and sampling

A total of 779 chicken product samples were collected, consisting of chicken meat (n = 683), giblets (n = 72), and processed chicken (*tataki*, thin slices of raw chicken with a minimally seared surface, n = 24). Of these samples, 622 samples originated from Japanese farms, and animals were slaughtered in Japan, 145 samples were from unrecorded origins, and 12 samples came from animals reared and slaughtered outside of Japan (11 samples were imported from Brazil, and one sample was from an unrecorded origin). All samples apart from two (described below) were collected in Fukuoka Prefecture, Japan, from 1996–2010. A total of 554 of the 779 samples were randomly collected from 282 retail outlets, including supermarkets and butcher shops, by Fukuoka prefectural food hygiene inspectors. A further 225 samples were randomly collected from 42 retail outlets by the authors from 2008–2010. Two of the 225 samples were collected in 2008 from two retail outlets in Nagasaki Prefecture, which neighbors Fukuoka Prefecture, while the remaining 223 samples were collected from 40 retail outlets in Fukuoka Prefecture, Japan.

The bacterial isolation method used for samples collected from 1996–1998 has been reported previously [16]. Isolations carried out from 1999–2010 were conducted in accordance with the method for isolating bacteria from meat samples described in our previous report [17], except for 85 samples collected in 2008. *Salmonella* isolation was carried out from these 85 samples using a previously described method [16] with a minor modification, as follows: Rappaport-Vassiliadis enrichment broth (Oxoid Ltd., Hampshire, UK) was used for the isolations, along with mannitol, lysine, crystal violet, brilliant green agar (Oxoid Ltd.) and Rimler-Shotts-Maeda agar (Kanto Chemical Co., Tokyo, Japan). The overall incidence of *Salmonella* isolation from the samples was 45.6% (355/779) (Table 1), with isolation rates of 45.5% (311/683), 54.2% (39/72), and 20.8% (5/24) from the chicken meat, giblets, and processed chicken samples, respectively. All salmonellae were isolated at the Fukuoka Institute of Health and Environmental Sciences, except for 52 isolates that were obtained from Nakamura Gakuen University Junior College in Fukuoka Prefecture. All the isolates were serotyped using somatic antisera and flagella antisera (Denka Seiken Co., Tokyo, Japan), as previously described [16].

**Table 1:** Number of tested chicken meat product samples, detected *Salmonella* isolates, resistant isolates, and resistance genes by year.

**Isolation year**	**1996**	**1997**	**1998**	**1999**	**2000**	**2001**	**2002**	**2003**
No. of samples tested	41	21	34	34	35	36	33	39
No. of *Salmonella*-positive samples	16	9	6	4	20	16	14	21
(%)	(39.0%)	(42.9%)	(17.6%)	(11.8%)	(57.1%)	(44.4%)	(42.4%)	(53.8%)
No. of *Salmonella* isolates	16	12	6	5	20	16	14	21
No. of samples from which multiple *Salmonella* serotypes were isolated	0	3	0	1	0	0	0	0
*Salmonella* serotypes isolated, (No. of isolate)	Corvallis (1)*,	Agona (1),	Corvallis (3),	Infantis (3),	Corvallis (1),	Haifa (2),	Infantis (12),	Cerro (1),
	Haifa (1),	Corvallis (1),	Enteritidis (1),	Untypeable with O7 (1),	Infantis (18),	Infantis (8),	Yovokome (1),	Haifa (1),
	Infantis (12),	Haifa (1),	Infantis (1),	Untypeable (1)	Untypeable with O7 (1)	Virchow (2),	Untypeable with O7:y:− (1)	Infantis (14),
	Typhimurium (1),	Infantis (5),	Typhimurium (1)			Untypeable with O18: Z4, Z23: − (3),		Manhattan (2),
	Untypeable with O4:i: (1)	Typhimurium (2),				Untypeable with O7 (1)		Untypeable (3)
		Untypeable with O4:b:− (1),						
		Untypeable with O8:z4,z24 (1)						
Untested isolates with antimicrobial susceptibility	-	-	-	-	-	-	-	-
No. of tested isolates	16	12	6	5	20	16	14	21
(No. of samples)	(16)	(9)	(6)	(4)	(20)	(16)	(14)	(21)
No. of resistant isolates	0	0	0	0	0	0	0	0
(Percentage of tested isolates)	(0.0%)	(0.0%)	(0.0%)	(0.0%)	(0.0%)	(0.0%)	(0.0%)	(0.0%)
Detected resistance genes and serotypes	-	-	-	-	-	-	-	-
Isolation year	2004	2005	2006	2007	2008	2009	2010	Total
No. of samples tested	35	40	40	40	157	88	106	779
No. of *Salmonella*-positive samples	13	22	13	22	76	48	55	355
(%)	(37.1%)	(55.0%)	(32.5%)	(55.0%)	(48.4%)	(54.5%)	(51.9%)	(45.6%)
No. of *Salmonella* isolates	13	25	14	24	81	50	61	378
No. of samples from which multiple *Salmonella* serotypes were isolated	0	3	1	2	4^†^	2	6	22
*Salmonella* serotypes isolated, (No. of isolate)	Enteritidis (1),	Enteritidis (1),	Dunkwa (1),	Corvallis (1),	Enteritidis (1),	Enteritidis (1),	Agona (1),	Infantis (180),
	Infantis (9),	Haifa (1),	Infantis (8),	Emek (1),	Eppendorf (2),	Infantis (14),	Infantis (20),	Schwarzengrund (70),
	Manhattan (1),	Corvallis (1),	Manhattan (3),	Enteritidis (1),	Infantis (33),	Manhattan (8),	Manhattan (19),	Manhattan (52),
	Yovokome (1),	Infantis (15),	Schwarzengrund (2)	Eppendorf (1),	Jamaica (1),	Schwarzengrund (21),	Schwarzengrund (18),	Untypeable (11),
	Untypeable (1)	Manhattan (2),		Infantis (8),	Manhattan (15),	Untypeable with O4 (3),	Virchow (1),	Corvallis (8),
		Montevideo (1),		Manhattan (2),	Schwarzengrund (21),	Untypeable (3)	Untypeable with O4 (1),	Enteritidis (6),
		Schwarzengrund (1),		Schwarzengrund (7),	Untypeable with O-untypeable:r:1,5 (3),		Untypeable (1)	Haifa (6),
		Typhimurium (1),		Typhimurium (1),	Unrecorded (5)			Typhimurium (6),
		Untypeable (2)		Untypeable with O4 (1),				Unrecorded (5),
				Untypeable with O7 (1)				and other serotypes^‡^
Untested isolates with antimicrobial susceptibility	-	-	-	-	Unrecorded (5)	-	-	5
No. of tested isolates	13	25	14	24	76	50	61	373
(No. of samples)	(13)	(22)	(13)	(22)	(71)	(48)	(55)	(350)
No. of resistant isolates	0	2	0	1	8	7	17	35
(Percentage of tested isolates)	(0.0%)	(8.0%)	(0.0%)	(4.2%)	(10.5%)	(14.0%)	(27.9%)	(9.4%)
Detected resistance genes and serotypes	-	CMY-2 (Infantis, n = 1)^§^,	-	CMY-2 (Infantis, n = 1)	CMY-2 (Infantis, n = 5),	CMY-2 (Infantis, n = 6),	CMY-2 (Infantis, n = 11),	CMY-2 (Infantis, n = 24),
		TEM-20 (Infantis, n = 1)			CMY-2 (O-untypeable:r:1,5, n = 1),	TEM-52 (Manhattan, n = 1)	CMY-2 (Manhattan, n = 1),	CTX-M-15 & TEM-1 (Manhattan, n = 2),
					TEM-52 (Infantis, n = 1),		TEM-52 (Manhattan, n = 1),	TEM-52 (Manhattan, n = 2),
					CTX-M-2 (Manhattan, n = 1)		CTX-M-2 (Infantis, n = 1)	CMY-2 (Manhattan, n = 1),
							CTX-M-15 & TEM-1 (Manhattan, n = 2),	CMY-2 (O-untypeable:r:1,5, n = 1),
							SHV-12 (Manhattan, n = 1)	CTX-M-2 (Infantis, n = 1),
								CTX-M-2 (Manhattan, n = 1),
								SHV-12 (Manhattan, n = 1),
								TEM-20 (Infantis, n = 1),
								TEM-52 (Infantis, n = 1)

*Corvallis, *Salmonella enterica* subsp. *enterica* serovar Corvallis.

^†^One sample contained three serotypes, while other multi-*Salmonella* samples contained two serotypes.

^‡^Other serotypes, untypeable with O4 (5), untypeable with O7 (4), Eppendorf (3), untypeable with O18: Z4, Z23: − (3), untypeable with O-untypeable:r:1,5 (3), Virchow (3), Agona (2), Yovokome (2), Cerro (1), Dunkwa (1), Emek (1), Jamaica (1), Montevideo (1), untypeable with O4:b:− (1), untypeable with O4:i: (1), untypeable with O7:y:− (1), anduntypeable with O8:z4,z24 (1).

^§^CMY-2, *bla*_CMY-2_.

A total of 378 distinct *Salmonella* isolates were collected from 355 of the 779 chicken product samples. Twenty-one and one samples yielded two and three distinct isolates, respectively (Table 1). Of the 378 *Salmonella* isolates, 186 (49.2%) were isolated over the first 12 years (1996–2007), while the remaining 192 (50.8%) isolates consisted of 81 (21.4%), 50 (13.2%), and 61 (16.1%) isolates collected in 2008, 2009, and 2010, respectively. Of the 378 isolates, 373 (from 350 samples) were examined in the following assays (Table 1). The remaining five isolates were not tested because of damage incurred during the storage process. The tested isolates were obtained from chicken meat (n = 310), giblets (n = 35), and processed chicken (n = 5) that had been collected from 195 different outlets. The susceptibility-tested *Salmonella* isolates constituted 16 serovars, along with 28 untypeable strains. The dominant serovars were *Salmonella enterica* subsp. *enterica* serovar (*S*.) Infantis (n = 180), *S.* Schwarzengrund (n = 70), and *S.* Manhattan (n = 52) (Table 1). The incidences of S*almonella* from the samples collected from 1996 to 1997, and those from 85 samples collected in 2008, have been reported previously [16,18].

### Antimicrobial susceptibility testing

Antimicrobial susceptibility testing was performed using the disk diffusion method according to the Clinical and Laboratory Standards Institute (CLSI) guidelines [19,20]. The following 11 antimicrobials were used: cefpodoxime (10 μg), cefotaxime (30 μg), ceftazidime (30 μg), cefepime (30 μg), cefoxitin (30 μg), cefmetazole (30 μg), cefotetan (30 μg), moxalactam (30 μg), imipenem (10 μg), meropenem (10 μg), and panipenem (10 μg). Disks containing these antimicrobials were purchased from Becton Dickinson (Franklin Lakes, NJ). *Escherichia coli* ATCC 25922 was used as a quality control strain in accordance with CLSI guidelines [19,20]. *Klebsiella pneumoniae* ATCC 700603 and *E. coli* ATCC 35218 were used as reference strains. We used the CLSI criteria for resistance breakpoints for further resistance gene detection analyses [21], except for panipenem, which we defined in this study (23 mm in diameter). Isolates that were resistant to one or more of these antimicrobials were then examined by PCR to determine the presence of β-lactamase genes (*bla*_CMY_, *bla*_CTX-M_, *bla*_TEM_, and *bla*_SHV_), as previously described (Table 2) [22–25]. The resistant isolates with a zone diameter less than or equal to the breakpoints for cefpodoxime, cefotaxime, ceftazidime, or cefepime were also examined to identify ESBL production, using the phenotypic confirmatory test [21] with cefotaxime (30 μg)-clavulanic acid (Becton Dickinson), ceftazidime (30 μg)-clavulanic acid (Becton Dickinson), and cefpodoxime (10 μg)-clavulanic acid (10 μg) (Nissui Pharmaceutical Co., Tokyo, Japan) disks.

**Table 2:** Sequences of primers used in this study.

***bla* gene**	**Purpose**	**Primer Name**	**Nucleotide Sequence 5′–3′**	**Reference**
*bla*_CMY_	Detection	cmy-F	GACAGCCTCTTTCTCCACA	[22]
		cmy-R	TGGAACGAAGGCTACGTA	[22]
	Sequencing	CMY2-outF	GTTACAATGTGTGAGAAGCAGTC	This study
		CMY2-outR	ATGGGATTTTCCTTGCTGTA	This study
		CMY2-R0	CAGTATTTCGTGACCGGA	This study
		CMY2-F3	CTGGATTACGGTTCCGCA	Thi0073 study
*bla*_CTX-M_	Detection and Sequencing	CTX-MU1	ATGTGCAGYACCAGTAARGT	[23]
		CTX-MU2	TGGGTRAARTARGTSACCAGA	[23]
	Sequencing	*bla*_CTX-M-1_, forward	CTTCCAGAATAAGGAATCCC	[26]
		*bla*_CTX-M-1_, reverse	CGTCTAAGGCGATAAACAAA	[26]
		CTX-outF2	GCCAAGGGATAATACTAATAGAGG	This study
		CTX-outR	GCGGAATGATAGAAAGAGATGAG	This study
		CTX-F2	ACAATACTGCCATGAATAAGCTG	This study
		CTX-R0	CAATCAGCTTATTCATGGCA	This study
*bla*_TEM_	Detection	MAb/F	GGGGAGCTCATAAAATTCTTGAAGAC	[24]
		MAb/R	GGGGGATCCTTACCAATGCTTAATCA	[24]
	Sequencing	MAb-F2	AGCCCTCCCGTATCGTAGTT	This study
		MAb-F1	GAGGACCGAAGGAGCTAACC	This study
		MAb-outR	AACTACGATACGGGAGGGCT	This study
*bla*_SHV_	Detection	SHV-F	AGGATTGACTGCCTTTTTG	[25]
		SHV-R	ATTTGCTGATTTCGCTCG	[25]
	Sequencing	SHV-forw	CAAAACGCCGGGTTATTC	[27]
		SHV-rev	TTAGCGTTGCCAGTGCT	[27]
		SHVseq-forw	GGATTGACTGCCTTTTTGC	[27]
		SHVseq-rev	GCAAAAAGGCAGTCAATCC	[27]

### DNA sequence analysis of resistance genes

Nucleotide sequences of the resistance genes were determined using the DNA sequencing primers listed in Table 2 [26,27]. PCR products were purified using Microcon filters (Millipore Corporation, Bedford, MA) and were sequenced using a BigDye Terminator Cycle Sequencing Reaction v. 3.1 kit (Applied Biosystems, Carlsbad, CA) on a 3500 Genetic Analyzer (Applied Biosystems). The complementary sense and antisense sequences were aligned using the SeqManII program within the Lasergene software package (DNASTAR, Madison, WI). The DNA sequences and deduced amino acid sequences were examined using the BLAST program at the DNA Data Bank of Japan (DDBJ) [28], and the “β-Lactamase Classification and Amino Acid Sequences for TEM, SHV, and OXA Extended-Spectrum and Inhibitor Resistant Enzymes” website [29].

### Pulsed-field gel electrophoresis and large plasmid profile analysis

*S.* Infantis isolates (n = 26), *S.* Manhattan isolates (n = 7), and one O-untypeable:r:1,5 isolate that were shown to carry resistance genes (*bla*_CMY_, *bla*_TEM_, *bla*_CTX-M_, and/or *bla*_SHV_) were further examined using pulsed-field gel electrophoresis (PFGE) profiling and large-plasmid profile (LPP) analysis. To compare isolates with and without resistance genes, *S*. Infantis isolates (n = 18) and *S.* Manhattan isolates (n = 18) showing susceptibility to 11 antimicrobials were also examined. Two, three, five, four, and four susceptible *S*. Infantis isolates were collected in 2006, 2007, 2008, 2009, and 2010, respectively. Of the 18 susceptible *S*. Manhattan isolates, two, one, two, three, two, two, three, and three were collected in 2003, 2004, 2005, 2006, 2007, 2008, 2009, and 2010, respectively. PFGE using *Bln*I restriction digestion was conducted as previously described [30]. Similarity and cluster analyses were performed using the Dice coefficients of similarity and an unweighted pair group method with average linkage, respectively, using FPQuest Software (Bio-Rad Laboratories, Hercules, CA). For LPP analysis, total DNA was treated with 2 U/ml of S1 nuclease (incubated at 37°C for 45 min), followed by PFGE separation [30].

### Detection of IS*Ecp1* and other genes related to *bla*_CMY-2_

Genetic variation in the regions upstream and downstream of *bla*_CMY-2_ was identified by restriction fragment length polymorphism (RFLP) and sequence analysis. Twenty-five *bla*_CMY-2_-harboring isolates (23 *S*. Infantis, one *S*. Manhattan, and one O-untypeable:r:1,5) were tested. A PCR fragment containing part of *bla*_CMY-2_ and its upstream region was generated by PCR using a forward primer located in IS*Ecp1* (ISEcp1-F: 5′-CTATCCGTACAAGGGAGTGT-3′) [31] and a reverse primer located in *bla*_CMY_ (cmy-R) (Table 2). A PCR fragment containing part of the *bla*_CMY-2_ gene and its downstream region was generated with a forward primer located in *bla*_CMY_ (cmy-F) (Table 2) and a reverse primer located in *sugE* (SugE-R: 5′- ATTGCAGGTTTGCTCGAAGT-3′). PCR was conducted using a HotStarTaq Plus Master Mix Kit (Qiagen, Hilden, Germany), and products were digested with *Mse*I (New England BioLabs, Ipswich, MA) at 37°C for 1 h. Digested fragments were separated by electrophoresis on 1% (w/v) agarose gels at 100 V for 30 min. Additionally, the PCR products of three *S.* Infantis isolates harboring *bla*_CMY-2_ (isolates 1993, 2127, and 2150) and one *S*. Manhattan isolate harboring *bla*_CMY-2_ (isolate 2179) were sequenced as described above. These three *S.* Infantis isolates were chosen for sequence analysis of the IS*Ecp1* region because they showed the dominant PFGE profiles (PFP) and LPP characteristics.

### Localization of *bla*_CMY-2_ by Southern blot analysis

To determine whether *bla*_CMY-2_ was located in the chromosome or on a plasmid, S1 nuclease (New England BioLabs) and *Bln*I (Takara Bio, Otsu, Japan) digestions were prepared from the total DNA of the four isolates selected for IS*Ecp1* analysis (harboring *bla*_CMY-2_) (*S*. Infantis isolates 1993, 2127, and 2150, and *S*. Manhattan isolate 2179), as well as two susceptible isolates (*S*. Infantis isolate 1737 and *S*. Manhattan isolates 2129) for use as *bla*_CMY-2_-negative controls. Total DNA was treated with 2 U/ml of S1 nuclease (incubated at 37°C for 45 min) or *Bln*I (incubated at 37°C for 16 h), followed by PFGE separation [30]. Separated fingerprints were transferred to positively-charged Nylon membranes (Roche Applied Science, Penzberg, Germany) and hybridized with PCR-generated *bla*_CMY-2_ digoxigenin-labeled probes (Roche Diagnostics, Basel, Switzerland) using hybridization solution (Roche Diagnostics) according to the manufacturer’s instructions and a previous report [32].

## Results

### Antimicrobial susceptibility testing, PCR and sequence analysis of *bla* genes, and extended-spectrum β-lactamase phenotyping

Of the 373 tested isolates, 35 isolates from 31 chicken meat and four giblet samples showed resistance to one or more antimicrobials. These 35 chicken product samples included 27 samples originating from Japanese farms and 8 samples from unrecorded origins. Testing showed that 35 (9.4%), 24 (6.4%), 19 (5.1%), 13 (3.5%), and four (1.1%) isolates were resistant to cefpodoxime, cefoxitin, cefotaxime, ceftazidime, and cefepime, respectively. No isolates were resistant to cefmetazole, cefotetan, moxalactam, imipenem, meropenem, or panipenem. All 35 resistant isolates harbored one or two of the resistance genes tested, with 26 pAmpC (*bla*_CMY-2_)-harboring isolates (24 *S*. Infantis, one *S*. Manhattan, and one O-untypeable:r:1,5) and nine ESBL-producing isolates, harboring *bla*_CTX-M_ (n = 4, consisting of two *bla*_CTX-M-2_ and two *bla*_CTX-M-15_), *bla*_TEM_ (n = 4, consisting of one *bla*_TEM-20_ and three *bla*_TEM-52_), and *bla*_SHV_ (n = 1, *bla*_SHV-12_) (Tables 1 and 3). Two of the nine ESBL isolates harbored the non-ESBL gene *bla*_TEM-1_ as well as *bla*_CTX-M-15_.

**Table 3:** Antimicrobial-resistance patterns, pulsed-field profiles, and plasmid profiles of resistance genes in *Salmonella enterica* subsp. *enterica* serovar (*S.*) Infantis, *S*. Manhattan, and *Salmonella* O-untypeable isolates.

**Serovars**	**Resistance genes detected**	Antimicrobial- resistance patterns*	**Pulsed-field profiles**	**Large plasmid profiles**	**No. of isolates**
*S*. Infantis^†^	*bla*_CMY-2_	CPDX-CFX	C	1	6
(n = 45)	(n = 24)		B	1	2
			A	1	1
			E	1	1
			G	1	1
			I	1	1
			Loss^‡^	Loss	1
		CPDX-CTX-CAZ-CFX	C	1	4
			D	1	1
		CPDX-CTX-CFX	C	1	2
			B	1	1
		CPDX-CAZ-CFX	F	1	1
		CPDX-CTX	J	1	1
		CPDX	I	1	1
	*bla*_CTX-M-2_	CPDX-CTX-CFPM	K	2	1
	*bla*_TEM-20_	CPDX	L	1	1
	*bla*_TEM-52_	CPDX-CTX	L	1	1
	−^§^ (n = 18)	No resistance detected	H	1	5
	-		H	3	1
	-		L	1	4
	-		N	1	3
	-		P	1	1
	-		P	4	1
	-		I	1	1
	-		M	1	1
	-		Q	1	1
*S.* Manhattan^†^	*bla*_CMY-2_	CPDX-CTX-CAZ-CFX	R	5	1
(n = 25)	*bla*_CTX-M-2_	CPDX-CTX-CFPM	R	7	1
	*bla*_CTX-M-15_& *bla*_TEM-1_	CPDX-CTX-CAZ-CFPM	R	8	2
	*bla*_TEM-52_	CPDX-CTX-CAZ	R	6	2
	*bla*_SHV-12_	CPDX-CTX-CAZ	R	6	1
	−^§^ (n = 18)	No resistance detected	R	6	17
			S	6	1
O-untypeable:r:1,5 (n = 1)	*bla*_CMY-2_	CPDX-CTX-CAZ-CFX	E	1	1

*Determined with CPDX, cefpodoxime; CTX, cefotaxime; CAZ, ceftazidime; CFPM, cefepime; CFX, cefoxitin; CTT, cefotetan; CMZ, cefmetazole; MOX, moxalactam; IPM, imipenem; MEPM, meropenem; and PAPM, panipenem.

^†^ *S*. Infantis, *Salmonella enterica* subsp. *enterica* serovar Infantis; *S.* Manhattan, *Salmonella enterica* subsp. *enteric*a serovar Manhattan.

^‡^Loss, an isolate that lost resistance during storage.

^§^These isolates were susceptible to all antimicrobial agents tested.

An analysis of the most common resistance genes harbored by each of the serotypes showed that *S*. Infantis with *bla*_CMY-2_ (n = 24) was the most common serotype/resistance gene combination. No other combination occurred in more than two isolates (Table 1, 3). Of the 26 isolates harboring *bla*_CMY-2_, 24 showed resistance to cefoxitin and two showed intermediate sensitivity to cefoxitin (15–17 mm), based on the CLSI criteria [21]. All 26 isolates harboring *bla*_CMY-2_ were susceptible to other cephamycins (cefmetazole, cefotetan, and moxalactam). Furthermore, six isolates harboring only *bla*_CMY-2_, which does not contribute to ESBL production, also showed ESBL phenotypes when tested with cefpodoxime-clavulanic acid (n = 5) or ceftazidime-clavulanic acid disks (n = 1).

Chicken product isolates harboring pAmpC and ESBL genes first appeared in 2005, and the percentage of resistant isolates increased significantly from 0% in 2004 to 27.9% in 2010 (*P* for trend = 0.006) (Table 1).

### Pulsed-field gel electrophoresis and large plasmid analysis

**Figure 1:**
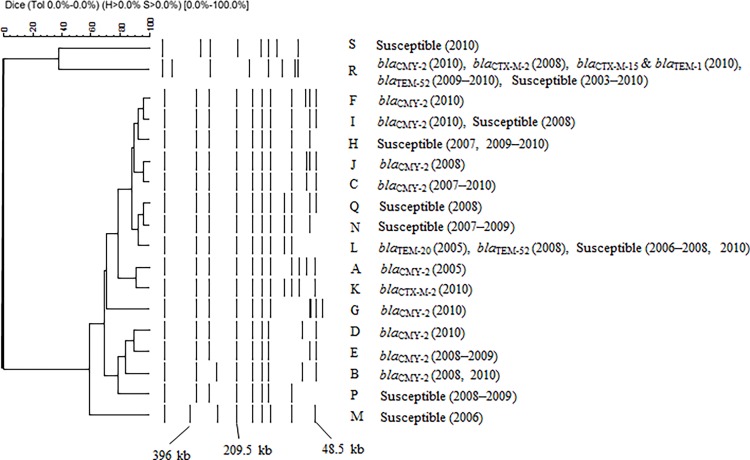
Pulsed-field gel electrophoresis profiles (PFPs) of 70 *Salmonella* isolates harboring *bla* and non-*bla* genes. The isolates consisted of 25 isolates harboring *bla*_CMY-2_ (23 *Salmonella enterica* subsp. *enterica* serovar Infantis, one *S*. Manhattan, and one O-untypeable:r:1,5), nine isolates harboring other *bla* genes (three *S*. Infantis and six *S.* Manhattan), and 36 isolates susceptible to 11 antibiotics (18 *S*. Infantis and 18 *S.* Manhattan). One *S*. Infantis isolate that harbored *bla*_CMY-2_ could not be analyzed by PFGE because of damage that occurred during storage. The letters, names, and figures in parentheses on the right of the dendrogram are PFPs, resistance genes, and sampling year of isolates, respectively. The 44 *S*. Infantis isolates were subtyped as: PFP A (n = 1), PFP B (n = 3), PFP C (n = 12), PFP D (n = 1), PFP E (n = 1), PFP F (n = 1), PFP G (n = 1), PFP H (n = 6), PFP I (n = 3), PFP J (n = 1), PFP K (n = 1), PFP L (n = 6), PFP M (n = 1), PFP N (n = 3), PFP P (n = 2), and Q (n = 1). The 25 *S.* Manhattan isolates belonged to subtypes PFP R (n = 24) and PFP S (n = 1). One O-untypeable:r:1,5 isolate was PFP E. The scale indicates the percent similarity, as determined by the Dice coefficients.

One *S*. Infantis isolate that harbored *bla*_CMY-2_ could not be subtyped by PFGE, LPP, and the following assays because of losses in resistance incurred during storage. Therefore, only 26 of 27 *S*. Infantis isolates that harbored *bla* genes (23 *bla*_CMY-2_, one *bla*_CTX-M-2_, one *bla*_TEM-20_, and one *bla*_TEM-52_) and 18 *S*. Infantis isolates showing susceptibility to the antimicrobials were tested (Table 3), along with the O-untypeable:r:1,5 and *S.* Manhattan isolates. PFPs are shown in Table 3 and Fig. 1. *S*. Infantis (n = 44) and O-untypeable:r:1,5 isolates (n = 1) generated 16 PFPs (A–Q, except for O), and *S*. Manhattan (n = 25) generated two PFPs (R and S). *S*. Infantis isolates carrying *bla*_CMY-2_ that were isolated in 2008 (n = 5), 2009 (n = 6), and 2010 (n = 10) generated three, two, and six PFPs, respectively. PFP C was the predominant PFP (n = 12) for *S.* Infantis isolates amongst the 16 PFPs. All isolates displaying PFP C harbored *bla*_CMY-2_. *S*. Manhattan carrying *bla*_CMY-2_ (n = 1) that was isolated in 2010 generated PFP R, as did the other 23 *S*. Manhattan isolates.

**Figure 2:**
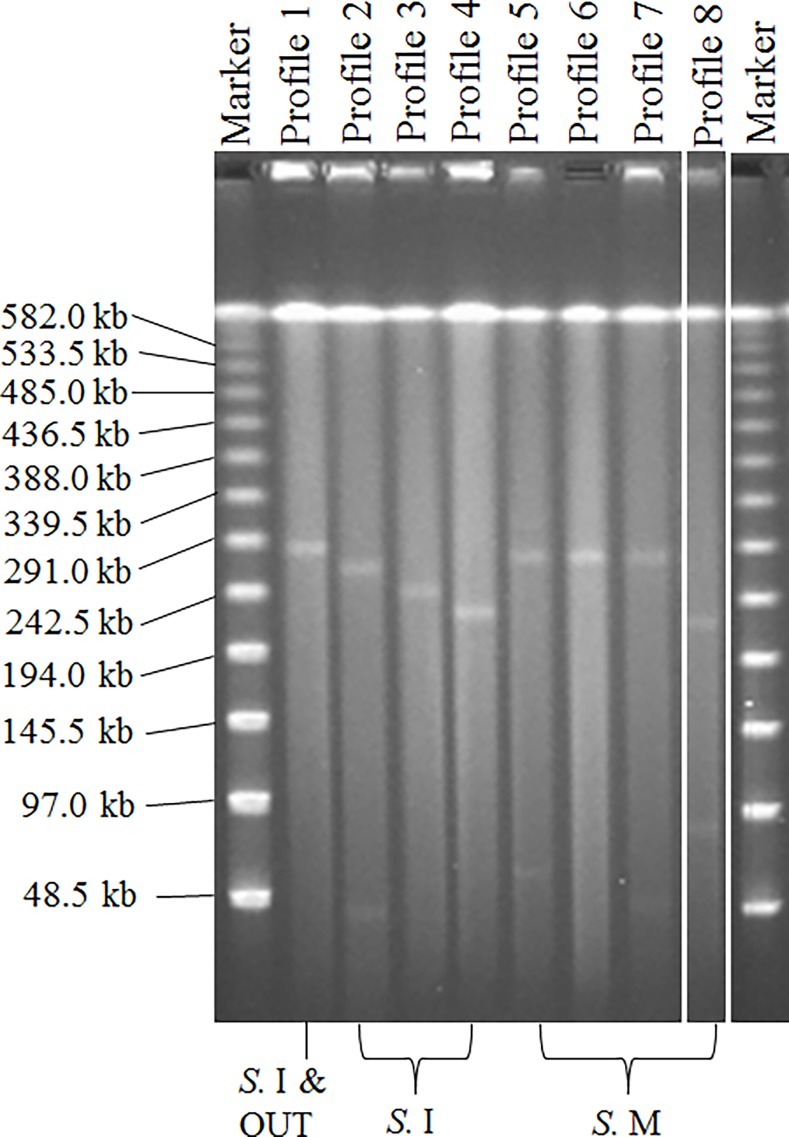
Large plasmid profiles of 70 *Salmonella* isolates. These isolates consisted of 25 isolates harboring *bla*_CMY-2_ (23 *Salmonella enterica* subsp. *enterica* serovar Infantis, one *S*. Manhattan, and one O-untypeable:r:1,5), nine isolates harboring other *bla* genes (three *S*. Infantis and six *S.* Manhattan), and 36 isolates susceptible to 11 antibiotics (18 *S*. Infantis and 18 *S.* Manhattan). The 70 isolates generated eight large plasmid profiles (LPPs). The *S.* Infantis isolates generated LPPs 1 (n = 41), 2 (n = 1), 3 (n = 1), and 4 (n = 1). All *S.* Infantis isolates harboring *bla* genes showed LPP 1, except for one *S*. Infantis isolate carrying *bla*_CTX-M-2_ that was classified as LPP 2. All 18 susceptible *S.* Infantis isolates showed LPP 1, except for two isolates that were collected in 2009 and 2008 showing LPP 3 and LPP 4, respectively. One O-untypeable:r:1,5 isolate was classified as LPP 1. *S.* Manhattan isolates generated four different LPPs (LPP 5-LPP 8). LPPs 5, 6, 7, and 8 were found in one, 21, one, and two *S.* Manhattan isolate(s), respectively. *S.* Infantis, O-untypeable:r:1,5, and *S.* Manhattan are expressed as *S.* I, OUT, and *S.* M in the figure, respectively.

LPPs are shown in Table 3 and Fig. 2. *S*. Infantis (n = 44) generated four different LPPs (LPP 1-LPP 4). Forty-one out of the 44 *S*. Infantis isolates, including *bla*_CMY-2_-positive isolates, were classified as LPP 1. One O-untypeable:r:1,5 isolate harboring *bla*_CMY-2_ was classified as LPP 1. *S*. Manhattan (n = 25) generated four different LPPs (LPP 5-LPP 8). One *S*. Manhattan isolate carrying *bla*_CMY-2_ was classified as LPP 5.

### Confirmation of IS*Ecp1* and other genes related to *bla*_CMY-2_

*Mse*I-RFLP profiles of the regions upstream and downstream of *bla*_CMY-2_ were identical in all 25 isolates harboring *bla*_CMY-2_. *S*. Infantis isolates 1993, 2127, and 2150, showing the dominant PFP C and LPP 1 characteristics, and *S.* Manhattan isolate 2179, the only *S.* Manhattan isolate harboring *bla*_CMY-2_, were also used for sequencing of the *bla*_CMY-2_ upstream and downstream regions and for Southern blot analysis. A 2,389-bp fragment containing the *bla*_CMY-2_ gene from the selected isolates was sequenced. Results showed that these fragments from *S.* Infantis and *S.* Manhattan were identical. The fragments contained partial IS*Ecp1, bla*_CMY-2,_ *blc,* and partial *sugE* genes. The IS*Ecp1* sequences from the four selected isolates were 100% identical to the sequence reported for pNF4565 (GenBank accession AY581207; 2,389/2,389 bp, 100%), a plasmid carrying *bla*_CMY-2_ from *S*. Typhimurium (isolated from a human in 1999) [33].

### Localization of *bla*_CMY-2_ by Southern blot analysis

**Figure 3:**
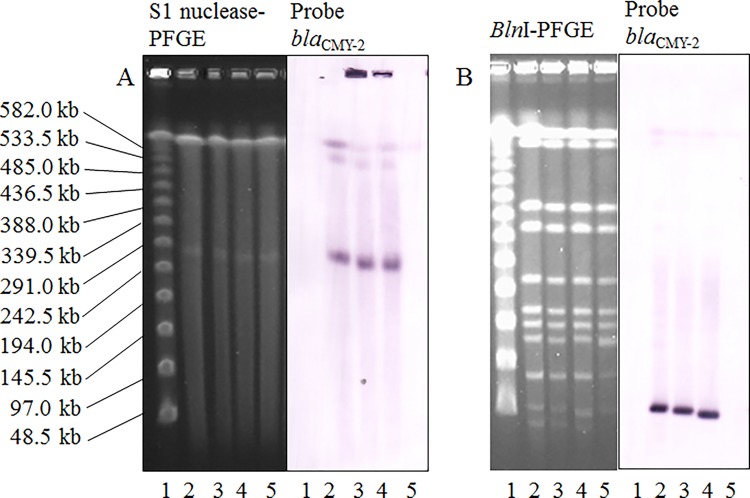
Pulsed-field gel electrophoresis (PFGE) and Southern hybridization images of *Salmonella enterica* subsp. *enterica* serovar Infantis. Selected *Salmonella enterica* subsp. *enterica* serovar (*S*.) Infantis isolates were selected to demonstrate the plasmid location of *bla*_CMY-2_. (A) PFGE separation of S1 nuclease-digested genomic DNA from selected *S.* Infantis isolates, followed by Southern hybridization with a *bla*_CMY-2_ probe. Lane 1, Lambda ladder marker; lane 2, isolate 1993; lane 3, isolate 2127; lane 4, isolate 2150; and lane 5, isolate 1737, which does not harbor *bla*_CMY-2_. (B) *Bln*I-digested whole-genomic DNA from selected *S.* Infantis isolates, followed by Southern hybridization with a *bla*_CMY-2_ probe. Lane 1, Lambda ladder marker; lane 2, isolate 1993; lane 3, isolate 2127; lane 4, isolate 2150; and lane 5, isolate 1737, which does not harbor *bla*_CMY-2_.

All the *S*. Infantis isolates harboring *bla*_CMY-2_ carried plasmids of approximately 280 kb, as did the *bla*_CMY-2_-negative *S.* Infantis isolate (isolate 1737, LPP 1) (Fig. 3). Southern blot analysis using a *bla*_CMY-2_ gene probe only produced a hybridization signal for the plasmids from the three *S*. Infantis isolates in which *bla*_CMY-2_ was detected by PCR (Fig. 3). As a result, these plasmids of approximately the same size were classified on the basis of the presence/absence of *bla*_CMY-2_. Southern hybridization using a *bla*_CMY-2_ gene probe with *Bln*I-digested genomic DNA from these isolates showed hybridization signals at approximately 54 kb in the three isolates harboring *bla*_CMY-2_.

**Figure 4:**
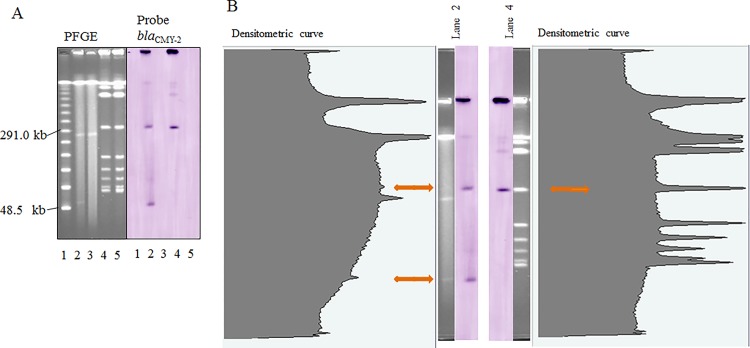
Location of *bla*_CMY-2_ in *Salmonella enterica* subsp. *enterica* serovar (*S*.) Manhattan. (A) Pulsed-field gel electrophoresis (PFGE) separation of S1 nuclease- or *Bln*I-digested genomic DNA from *S.* Manhattan isolates, followed by Southern hybridization with a *bla*_CMY-2_ probe. Lane 1, Lambda ladder marker; lanes 2 and 4, isolate 2179, which harbors *bla*_CMY-2_; lanes 3 and 5, isolate 2129, which does not harbor *bla*_CMY-2_. Lanes 2 and 3, S1 nuclease-digested genomic DNA; lanes 4 and 5, *Bln*I-digested genomic DNA. (B) Densitometric curves of PFGE separation with S1 nuclease- and *Bln*I-digested genomic DNA from *S.* Manhattan isolate 2179. The arrows show hybridization signals and corresponding positions of densitometric curves. Lambda ladder marker consisted of concatemers starting at 48.5 kb.

One *S*. Manhattan isolate that contained *bla*_CMY-2_ showed two hybridization signals (approximately 310 kb and 60 kb) (Fig. 4). The 310-kb hybridization signal appeared to be hybridization background; however, the point was confirmed as a small peak of density using densitometric analysis (FPQuest Software, Bio-Rad Laboratories) (Fig. 4). Therefore, the *S*. Manhattan isolate was confirmed to harbor two *bla*_CMY-2_ genes in putative plasmids of 310-kb and 60-kb in size. No hybridization signal was observed for the *bla*_CMY-2_-negative *S*. Manhattan isolate. Southern blot analysis of the *S*. Manhattan isolate was carried out on two separate occasions, with the same result obtained each time.

## Discussion

In the present study, pAmpC (*bla*_CMY-2_)-harboring and ESBL-producing (resulting from carriage of *bla*_CTX-M-2_, *bla*_CTX-M-15_, *bla*_TEM-20_, *bla*_TEM-52_, or *bla*_SHV-12_) *Salmonella* were isolated from chicken product samples collected after 2005 in Japan. The incidence of *Salmonella* in these samples was 45.6%, showing that *Salmonella* contamination of chicken products is very common in this region of Japan. The percentage of resistant isolates also increased significantly from 0% in 2004 to 27.9% in 2010. This can be attributed in part to the increase in the number of *S*. Infantis isolates harboring *bla*_CMY-2_. We determined that the *bla*_CMY-2_ gene was located close to an IS*Ecp1* element in an approximately 280-kb plasmid, pAmpC, in *S*. Infantis. The presence of pAmpC in *S*. Infantis from chicken meat and giblets is a significant public health concern because *S.* Infantis is one of the main causes of human salmonellosis [34], and chicken meat is the main source of human *S.* Infantis infection in Japan [35].

Interestingly, Taiwan is often the first Asian country in which CMY-2-type β-lactamase-producing bacteria are isolated from humans. Fourteen, two, and one human *Salmonella* isolates harboring *bla*_CMY-2_ were isolated in Taiwan in 2000–2002, in Korea in 2002, and in Japan in 2007, respectively [1,36,37]. In the case of other *Enterobacteriaceae*, six, 12, and 17 human *E. coli* isolates harboring *bla*_CMY-2_ were isolated in Taiwan in 1999, in China in 2003–2005, and in Japan in 2007–2009, respectively [38–40]. Moreover, 32, 21, and one human *K. pneumoniae* isolates harboring *bla*_CMY-2_ were also isolated in Taiwan in 1999–2002 [41], in Korea in 2002–2003 [42], and in China in 2004 [39], respectively. The reason for this apparent first emergence of *bla*_CMY-2_-carrying isolates in Taiwan is unknown, and may just be a result of differences in monitoring and reporting protocols; however, this phenomenon warrants further enquiry considering the international circulation of livestock and their products, especially chickens.

The first *Salmonella* harboring *bla*_CMY-2_ were isolated from chicken meat in Japan, and were reported in the mid-2000s by Taguchi et al. [15]. The isolation years (2004 or 2005, exact date unknown) of the Taguchi study coincided with the first year of detection (2005) of CMY-2 β-lactamase-producing *Salmonella* in the present study. Therefore, it could be surmised that *bla*_CMY-2_-harboring *Salmonella* began to appear in chicken meat in Japan from around 2004. Interestingly, Hiki et al. also reported that the incidence of *bla*_CMY-2_-containing *E. coli* isolated from broiler chickens in Japan increased after 2004 [43]. CMY-2 β-lactamase-producing *E. coli* from chicken meat were reported in Taiwan in 2002 [44], but were not reported in Japan until 2006 [45,46]. The possibility of interspecies transmission of *bla*_CMY-2_ between *Salmonella* and other *Enterobacteriaceae* populations like *E. coli* in Japanese chicken birds cannot be denied. However, Shahada et al. reported that no interspecies transmission was detected in Japanese broilers when they compared *Salmonella* and *E. coli* isolates from Japanese chickens in 2010 and 2011 [47]. Therefore, further research is required to determine when and where the interspecies transmission of *bla*_CMY-2_ from other *Enterobacteriaceae* to *Salmonella* is occurring.

The presence of *bla*_CMY-2_ genes on both the putative 310-kb plasmid and the 60-kb plasmid in the *S*. Manhattan isolate identified in this study might play a role in the relatively strong ESC-resistance of this isolate. The inhibition zone diameters for cefotaxime and ceftazidime (16 and 12 mm, respectively) for this isolate were the smallest of all 26 isolates harboring *bla*_CMY-2_ (averaged 23 mm and 18 mm, respectively). In addition, the zone diameters for cefpodoxime and cefoxitin for the *S*. Manhattan isolate were also amongst the smallest for the 26 isolates. Again, the presence of two copies of the *bla*_CMY-2_ gene in this isolate may contribute to this high level of resistance; however, further studies are needed to confirm the presence and structure of the putative 310-kb plasmid.

In this study, a 2,389-bp fragment surrounding *bla*_CMY-2_ was identical to a partial sequence of previously reported plasmids, including pNF4656 (*S*. Newport, >100 kb) [33], pNF1358 (*S*. Typhimurium, >100 kb) [33], pCVM29188 (*S*. Kentucky, 101,461 bp) [48], and pUMNK88 161 (*E. coli*, 161,081 bp) [49]. These studies reported that the mobile element IS*Ecp1* in the plasmids led to acquisition of various resistance genes in addition to *bla*_CMY-2_. As *S.* Infantis plasmids from both the resistant and susceptible isolates (Fig. 2) were indistinguishable in size, the transfer of *bla*_CMY-2_ between these plasmids should be studied.

The present study suggests some flaws in common screening approaches for ESBL detection. We identified an isolate carrying *bla*_TEM-20_ that was only resistant to cefpodoxime and cefepime. Such a strain could escape detection by traditional ESBL tests, which use only cefotaxime and ceftazidime [1,10,50]. Furthermore, this isolate showed intermediate sensitivity (20 mm) to ceftiofur (30 μg) (data not shown), which supports previous findings by Rodriguez et al. [11]. Therefore, routine ESBL screening may be improved by the use of cefpodoxime, cefepime, and/or ceftiofur.

In conclusion, we found that *S.* Infantis isolates harboring plasmid-borne *bla*_CMY-2_ were prevalent in chicken products in Japan after 2005. None of the studied isolates showed resistance to carbapenem antibiotics; however, the dissemination of ESC-resistant *Salmonella* containing plasmid-mediated *bla*_CMY-2_ demands the development of continuous monitoring strategies in the interests of public health.
