# Correction: Increase in Resistance to Extended-Spectrum Cephalosporins in *Salmonella* Isolated from Retail Chicken Products in Japan

**DOI:** 10.1371/journal.pone.0121469

**Published:** 2015-03-23

**Authors:** The PLOS ONE Staff

There are a number of errors in Table 1. Please see the corrected Table 1 here.

**Table 1:** Number of tested chicken meat product samples, detected *Salmonella* isolates, resistant isolates, and resistance genes by year.

Isolation year	1996	1997	1998	1999	2000	2001	2002	2003
No. of samples tested	41	21	34	34	35	36	33	39
No. of *Salmonella*-positive samples	16	9	6	4	20	16	14	21
(%)	(39.0%)	(42.9%)	(17.6%)	(11.8%)	(57.1%)	(44.4%)	(42.4%)	(53.8%)
No. of *Salmonella* isolates	16	12	6	5	20	16	14	21
No. of samples from which multiple *Salmonella* serotypes were isolated	0	3	0	1	0	0	0	0
*Salmonella* serotypes isolated, (No. of isolate)	Corvallis (1)*,	Agona (1),	Corvallis (3),	Infantis (3),	Corvallis (1),	Haifa (2),	Infantis (12),	Cerro (1),
	Haifa (1),	Corvallis (1),	Enteritidis (1),	Untypeable with O7 (1),	Infantis (18),	Infantis (8),	Yovokome (1),	Haifa (1),
	Infantis (12),	Haifa (1),	Infantis (1),	Untypeable (1)	Untypeable with O7 (1)	Virchow (2),	Untypeable with O7:y:- (1)	Infantis (14),
	Typhimurium (1),	Infantis (5),	Typhimurium (1)			Untypeable with O18: Z4, Z23: —(3),		Manhattan (2),
	Untypeable with O4:i: (1)	Typhimurium (2),				Untypeable with O7 (1)		Untypeable (3)
		Untypeable with O4:b:- (1),						
		Untypeable with O8:z4,z24 (1)						
Untested isolates with antimicrobial susceptibility	-	-	-	-	-	-	-	-
No. of tested isolates	16	12	6	5	20	16	14	21
(No. of samples)	(16)	(9)	(6)	(4)	(20)	(16)	(14)	(21)
No. of resistant isolates	0	0	0	0	0	0	0	0
(Percentage of tested isolates)	(0.0%)	(0.0%)	(0.0%)	(0.0%)	(0.0%)	(0.0%)	(0.0%)	(0.0%)
Detected resistance genes and serotypes	-	-	-	-	-	-	-	-
Isolation year	2004	2005	2006	2007	2008	2009	2010	Total
No. of samples tested	35	40	40	40	157	88	106	779
No. of *Salmonella*-positive samples	13	22	13	22	76	48	55	355
(%)	(37.1%)	(55.0%)	(32.5%)	(55.0%)	(48.4%)	(54.5%)	(51.9%)	(45.6%)
No. of *Salmonella* isolates	13	25	14	24	81	50	61	378
No. of samples from which multiple *Salmonella* serotypes were isolated	0	3	1	2	4^†^	2	6	22
*Salmonella* serotypes isolated, (No. of isolate)	Enteritidis (1),	Enteritidis (1),	Dunkwa (1),	Corvallis (1),	Enteritidis (1),	Enteritidis (1),	Agona (1),	Infantis (180),
	Infantis (9),	Haifa (1),	Infantis (8),	Emek (1),	Eppendorf (2),	Infantis (14),	Infantis (20),	Schwarzengrund (70),
	Manhattan (1),	Corvallis (1),	Manhattan (3),	Enteritidis (1),	Infantis (33),	Manhattan (8),	Manhattan (19),	Manhattan (52),
	Yovokome (1),	Infantis (15),	Schwarzengrund (2)	Eppendorf (1),	Jamaica (1),	Schwarzengrund (21),	Schwarzengrund (18),	Untypeable (11),
	Untypeable (1)	Manhattan (2),		Infantis (8),	Manhattan (15),	Untypeable with O4 (3),	Virchow (1),	Corvallis (8),
		Montevideo (1),		Manhattan (2),	Schwarzengrund (21),	Untypeable (3)	Untypeable with O4 (1),	Enteritidis (6),
		Schwarzengrund (1),		Schwarzengrund (7),	Untypeable with O-untypeable:r:1,5 (3),		Untypeable (1)	Haifa (6),
		Typhimurium (1),		Typhimurium (1),	Unrecorded (5)			Typhimurium (6),
		Untypeable (2)		Untypeable with O4 (1),				Unrecorded (5),
				Untypeable with O7 (1)				and other serotypes^‡^
Untested isolates with antimicrobial susceptibility	-	-	-	-	Unrecorded (5)	-	-	5
No. of tested isolates	13	25	14	24	76	50	61	373
(No. of samples)	(13)	(22)	(13)	(22)	(71)	(48)	(55)	(350)
No. of resistant isolates	0	2	0	1	8	7	17	35
(Percentage of tested isolates)	(0.0%)	(8.0%)	(0.0%)	(4.2%)	(10.5%)	(14.0%)	(27.9%)	(9.4%)
Detected resistance genes and serotypes	-	CMY-2 (Infantis, n = 1)^§^,	-	CMY-2 (Infantis, n = 1)	CMY-2 (Infantis, n = 5),	CMY-2 (Infantis, n = 6),	CMY-2 (Infantis, n = 11),	CMY-2 (Infantis, n = 24),
		TEM-20 (Infantis, n = 1)			CMY-2 (O-untypeable:r:1,5, n = 1),	TEM-52 (Manhattan, n = 1)	CMY-2 (Manhattan, n = 1),	CTX-M-15 & TEM-1 (Manhattan, n = 2),
					TEM-52 (Infantis, n = 1),		TEM-52 (Manhattan, n = 1),	TEM-52 (Manhattan, n = 2),
					CTX-M-2 (Manhattan, n = 1)		CTX-M-2 (Infantis, n = 1)	CMY-2 (Manhattan, n = 1),
							CTX-M-15 & TEM-1 (Manhattan, n = 2),	CMY-2 (O-untypeable:r:1,5, n = 1),
							SHV-12 (Manhattan, n = 1)	CTX-M-2 (Infantis, n = 1),
								CTX-M-2 (Manhattan, n = 1),
								SHV-12 (Manhattan, n = 1),
								TEM-20 (Infantis, n = 1),
								TEM-52 (Infantis, n = 1)

*Corvallis, *Salmonella enterica* subsp. *enterica* serovar Corvallis.

^†^One sample contained three serotypes, while other multi-*Salmonella* samples contained two serotypes.

^‡^Other serotypes, untypeable with O4 (5), untypeable with O7 (4), Eppendorf (3), untypeable with O18: Z4, Z23: —(3), untypeable with O-untypeable:r:1,5 (3), Virchow (3), Agona (2), Yovokome (2), Cerro (1), Dunkwa (1), Emek (1), Jamaica (1), Montevideo (1), untypeable with O4:b:- (1), untypeable with O4:i: (1), untypeable with O7:y:- (1), and untypeable with O8:z4,z24 (1).

^§^CMY-2, *bla*_CMY-2_.

In Table 2, cells including “Thi0073 study” in the Reference column should read “This study”. Please see the corrected Table 2 here.

**Table 2:** Sequences of primers used in this study.

*bla* gene	Purpose	Primer Name	Nucleotide Sequence 5′–3′	Reference
*bla*_CMY_	Detection	cmy-F	GACAGCCTCTTTCTCCACA	[22]
		cmy-R	TGGAACGAAGGCTACGTA	[22]
	Sequencing	CMY2-outF	GTTACAATGTGTGAGAAGCAGTC	This study
		CMY2-outR	ATGGGATTTTCCTTGCTGTA	This study
		CMY2-R0	CAGTATTTCGTGACCGGA	This study
		CMY2-F3	CTGGATTACGGTTCCGCA	This study
*bla*_CTX-M_	Detection and Sequencing	CTX-MU1	ATGTGCAGYACCAGTAARGT	[23]
		CTX-MU2	TGGGTRAARTARGTSACCAGA	[23]
	Sequencing	*bla*_CTX-M-1_, forward	CTTCCAGAATAAGGAATCCC	[26]
		*bla*_CTX-M-1_, reverse	CGTCTAAGGCGATAAACAAA	[26]
		CTX-outF2	GCCAAGGGATAATACTAATAGAGG	This study
		CTX-outR	GCGGAATGATAGAAAGAGATGAG	This study
		CTX-F2	ACAATACTGCCATGAATAAGCTG	This study
		CTX-R0	CAATCAGCTTATTCATGGCA	This study
*bla*_TEM_	Detection	MAb/F	GGGGAGCTCATAAAATTCTTGAAGAC	[24]
		MAb/R	GGGGGATCCTTACCAATGCTTAATCA	[24]
	Sequencing	MAb-F2	AGCCCTCCCGTATCGTAGTT	This study
		MAb-F1	GAGGACCGAAGGAGCTAACC	This study
		MAb-outR	AACTACGATACGGGAGGGCT	This study
*bla*_SHV_	Detection	SHV-F	AGGATTGACTGCCTTTTTG	[25]
		SHV-R	ATTTGCTGATTTCGCTCG	[25]
	Sequencing	SHV-forw	CAAAACGCCGGGTTATTC	[27]
		SHV-rev	TTAGCGTTGCCAGTGCT	[27]
		SHVseq-forw	GGATTGACTGCCTTTTTGC	[27]
		SHVseq-rev	GCAAAAAGGCAGTCAATCC	[27]
